# The role of collaborative, multistakeholder partnerships in reshaping the health management of patients with noncommunicable diseases during and after the COVID-19 pandemic

**DOI:** 10.1007/s40520-021-01922-y

**Published:** 2021-07-28

**Authors:** Alessandro Monaco, Amaia Casteig Blanco, Mark Cobain, Elisio Costa, Nick Guldemond, Christine Hancock, Graziano Onder, Sergio Pecorelli, Mitchell Silva, Jos Tournoy, Caterina Trevisan, Mariano Votta, John Yfantopoulos, Stecy Yghemonos, Vincent Clay, Franco Mondello Malvestiti, Karine De Schaetzen, Georgia Sykara, Shaantanu Donde

**Affiliations:** 1 HEC, Rue de la Libération 1, 78350 Paris, Jouy-en-Josas France; 2 Grupo OAT Therapeutic Adherence Observatory, Observatorio de La Salud S.L, Madrid, Spain; 3 Younger Lives Ltd, London, UK; 4 Imperial College London, London, UK; 5 UCIBIO/REQUIMTE Applied Molecular Biosciences Unit, Faculty of Pharmacy and Competences Center On Active and Healthy Ageing (Porto4Ageing), University of Porto, Porto, Portugal; 6 Leiden University Medical Center LUMC-NL, Leiden, The Netherlands; 7 Sechenov-Russia/ I.M. Sechenov First Moscow State Medical University, Moscow, Russia; 8 C3 Collaborating for Health, London, UK; 9 Italian National Institute of Health, Istituto Superiore Di Sanità, Rome, Italy; 10 University of Brescia, Brescia, Italy; 11 Esperity, Brussels, Belgium; 12 KU, Leuven, Belgium; 13 Geriatric Unit, Department of Medicine, University of Padua, Padua, Italy; 14 Active Citizenship Network, Cittadinanzattiva APS, Rome, Italy; 15 National and Kapodistrian University of Athens, Athens, Greece; 16 Eurocarers, Brussels, Belgium; 17 Upjohn (Pfizer), Brussels, Belgium; 18 Viatris, Rome, Italy; 19 Viatris, Brussels, Belgium; 20 Viatris, Athens, Greece; 21 Viatris, London, UK

**Keywords:** Noncommunicable diseases, NCD, COVID-19, SARS-CoV-2, Patient advocacy, Informal carers, Ageing, Patient empowerment, Public–private partnerships, Healthcare, Adherence

## Abstract

**Background:**

Policies to combat the COVID-19 pandemic have disrupted the screening, diagnosis, treatment, and monitoring of noncommunicable (NCD) patients while affecting NCD prevention and risk factor control.

**Aims:**

To discuss how the first wave of the COVID-19 pandemic affected the health management of NCD patients, identify which aspects should be carried forward into future NCD management, and propose collaborative efforts among public–private institutions to effectively shape NCD care models.

**Methods:**

The NCD Partnership, a collaboration between Upjohn and the European Innovation Partnership on Active and Healthy Ageing, held a virtual Advisory Board in July 2020 with multiple stakeholders; healthcare professionals (HCPs), policymakers, researchers, patient and informal carer advocacy groups, patient empowerment organizations, and industry experts.

**Results:**

The Advisory Board identified barriers to NCD care during the COVID-19 pandemic in four areas: lack of NCD management guidelines; disruption to integrated care and shift from hospital-based NCD care to more community and primary level care; infodemics and a lack of reliable health information for patients and HCPs on how to manage NCDs; lack of availability, training, standardization, and regulation of digital health tools.

**Conclusions:**

Multistakeholder partnerships can promote swift changes to NCD prevention and patient care. Intra- and inter-communication between all stakeholders should be facilitated involving all players in the development of clinical guidelines and digital health tools, health and social care restructuring, and patient support in the short-, medium- and long-term future. A comprehensive response to NCDs should be delivered to improve patient outcomes by providing strategic, scientific, and economic support.

## Introduction

Non-communicable diseases (NCDs) account for more than half of the global burden of disease and are a major cause of mortality [1, 2]. In a recent report, the NCD Countdown 2030 collaborators highlighted that health policies that target reductions in blood pressure, smoking, and alcohol use are needed to substantially reduce NCD mortality [1]. Reducing premature mortality from the four major NCDs (cardiovascular diseases, cancers, chronic respiratory diseases, and diabetes) by one-third is estimated to have substantial effects on longevity [3]. In addition, equitable access to high-quality, effective prevention and treatment for acute and chronic NCDs is required [1]. Prevention of NCDs, as well as the management of NCD patients’ symptoms and disease, are both relevant for preventing negative outcomes associated with chronic disorders, including dependency, disability, mortality, and healthcare costs. However, the control policies for COVID-19 have already disrupted screening, diagnosis, treatment, and surveillance of NCD patients [4]. Thus, the COVID-19 pandemic has triggered multiple problems for individuals with NCDs and their informal carers. First, people with preexisting NCDs are more likely to suffer severe consequences of COVID-19, including a higher risk of hospitalization, complications, and mortality [5–9]. Second, the infection-control measures led to difficulties in accessing adequate treatment, medication, and care for NCDs, e.g., non-urgent outpatient visits were cancelled due to the risk of SAR-CoV-2 infection to patients and healthcare providers (HCPs), care avoidance due to fear of infection [10], and resources were reallocated for testing and treatment of COVID-19. The reduction of outpatient services has important treatment implications for patients who have preexisting NCD diagnoses and the family members who may provide unpaid care to them at home (informal carers), and it also affects prevention, screening, and diagnosis of new NCD cases. For example, a US study on screening and control of diabetes and dyslipidemia reported changes in outpatient services during February and March 2020, specifically an 81–90% fall in the rate of testing (LDL cholesterol and HbA1c testing) and a 52–60% drop in new medication therapy (statin and metformin therapy) [11] while another study reported a significant increase in severe diabetes foot complications such as amputations during the pandemic, which may affect quality of life, mortality, and morbidity [12]. Finally, HCPs were often unprepared to cope with this unprecedented health emergency, especially the remote monitoring of NCD patients, which may lead to inequalities in NCD care between patients according to setting, country, and type of disorder.

Many countries are finding it difficult to cope with the crisis because of new and pre-existing constraints in manpower and financing, both for the prevention and treatment of COVID-19 but also for the management of NCD patients. The pandemic is still ongoing, and some countries have already started new phases of lockdown and strict social distancing to cope with an increase in COVID-19 cases. Future waves are also likely to regularly reoccur while vaccine programs are ongoing. Therefore, there is a need to reshape and transform NCD care in the context of resilient and sustainable health and social systems both in the long-term (post-pandemic periods) and in the medium-term (when new waves of COVID-19 might lead to new lockdowns or shielding initiatives for NCD patients). This is also relevant for other emergency situations such as future natural disasters and other pandemics. Despite many difficulties, the COVID-19 emergency has generated a momentum in the collaborative approaches among public and private institutions to identify and implement effective therapeutic approaches for NCD patients. Multistakeholder collaborations bring together the public sector (e.g., policymakers, HCPs, researcher institutes), the private sector (e.g., industry, pharmaceutical companies, etc.), and civil society (e.g., community groups, patient and carer advocacy organizations etc.). All stakeholders have varying roles and capabilities that can be utilized to provide a comprehensive response to NCDs, which is important due to the multisectoral nature of NCDs [13, 14].

In the current paper, we present the output of a multistakeholder Advisory Board by the NCD Partnership on the topic of how to reshape NCD healthcare during and after the COVID-19 pandemic. The aim is to discuss how the first wave of the COVID-19 pandemic affected the health management of NCD patients and which aspects should be carried forward into the future management of NCDs, as well as to identify how collaborative efforts among public–private institutions can effectively shape NCD care models and improve patient outcomes by providing strategic, scientific, and economic support.

## Methods

The NCD Partnership (PARTners in Ncds Engage foR building Strategies to improve Healthy ageing In Patients) is an initiative that brings together different stakeholders from the public and private sector, who work together to find solutions to increase healthy ageing and reduce the burden of NCDs. It involves a collaboration between Upjohn and the European Innovation Partnership (EIP) on Active and Health Ageing (AHA) and unites multiple stakeholders including HCPs, policymakers, researchers, patient and carer advocacy groups, patient empowerment organizations, and industry experts. The NCD Partnership was formed in 2021 at the start of the COVID-19 pandemic and on 7-8^th^ July 2020, Upjohn hosted a virtual conference to identify barriers and challenges to NCD management during the COVID-19 pandemic. The current article provides output from the roundtable discussion and represents the perspectives of multiple stakeholders. The congress was held in the summer of 2020 and reflects the experts’ opinions based on their experience of the first wave of the COVID-19 pandemic, specifically during peak periods.

## Results

Table 1 depicts the main findings of the Advisory Board, in terms of issues and barriers faced by NCD patients and HCPs as a result of the COVID-19 pandemic, possible solutions for the future, and the role that multistakeholder partnerships can play, as described in more detail below.

**Table 1:** Issues and barriers faced by NCD patients, their informal carers, and HCPs during the first phase of the pandemic, solutions for the future, and the role of multistakeholder partnerships

	Issues and barriers	Solutions for the future	Role of multistakeholder partnerships
NCD management guidelines	Existing NCD protocols were not designed for NCD care during the pandemic period	Develop specific guidelines for management of NCD patients during the COVID-19 pandemic period and other potential future health system threats, specific to different scenarios/public health measures	Provide common platforms for policy makers and HCPs to work together to provide practical, timely guidelines
	Inadequate scientific information to HCPs on how to manage NCD patients	Establish research grants to increase evidence-based information on the best methods for NCD management during lockdown/shielding	Organize conferences / advisory boards to collect perspective of multiple stakeholders
			Facilitate the harmonization and dissemination of scientific evidence through structured collaboration and consistent dissemination programs
Integrated care	Shift from hospital-based NCD care to more primary based community-level care	Reshape the system by decentralizing care and shifting more NCD care into the community	Develop and implement tools that can help prevent patients having direct physical contact with HCPs while still undergoing high-quality healthcare services
	Disruption of integrated care	Increase patient self-support, aided by HCPs and informal carers, so that patients can self-manage their disease and symptoms	Promote infrastructures and competences that could allow community HCPs to intercept and address chronic patients' needs while guaranteeing a consistent remote monitoring also through the use of digital tools that empower patients to self-manage their disease and symptoms
		Define an active coordinator of care/increase use of care managers, actively trained for future pandemics	
Health information	Inadequate information to patients/carers on how to manage NCDs without direct support from HCPs	Produce tools to guide people to find appropriate, trustworthy scientific information to improve patient empowerment	Create information platforms for patients to view reliable information, led by independent experts, endorsed by patient advocacy groups and institutions
	Infodemics and misinformation	Develop “best practice” patient guidebooks or information platforms with reliable information sources	Provide private industry sponsorship to HCPs and researchers that can help to develop information that is endorsed and publicized by patient advocacy groups
		Produce practical tools to guide people to scientific information written in layman terms	
Digital health solutions	Digital health solutions were not standardized and largely unregulated	Co-develop digital solutions with the patient, with clear, transparent criteria on what constitutes a safe and effective solution Adapt already existing tools, modified to different contexts and different patient types	Provide an organizational structure and competences to foster the development of innovative solutions by pulling together key private and public stakeholders
	Uncertainty about which patients should be prioritized for telemedicine	Improve digital health literacy (training programs, education, tools focused on specific patient groups and people with specific needs)	Validate, harmonize, and conduct cost-effectiveness analysis to ensure that new technologies really generate an added value for the community
	Patients, carers, & HCPs were unprepared or lacked training	Define standards of care for technological solutions	Incentivize the use of effective solutions in HCPs, patients, and informal carers

### NCD management guidelines

#### Barriers and issues

At the start of the COVID-19 pandemic, many HCPs suffered from a lack of guidance on how to manage NCD care at all stages of the care spectrum, including prevention and risk factor control, diagnosis of new cases, and routine treatment of NCD patients. Existing NCD protocols were understandably not developed for the care environment that arose during the pandemic, where non-urgent, face-to-face consultations were cancelled. As the pandemic is a novel, worldwide phenomenon that has never been faced before, there was inadequate scientific information available for HCPs on how to manage NCDs and non-acute patients during the pandemic. Many HCPs were restricted in methods to communicate with patients and collaborate with their colleagues. Due to a lack of readiness and regulations on digital health solutions, discussed later, many HCPs tried to find solutions to deal with patients remotely using phone or video calls. Further, there were no clear strategies for managing individuals living in long-term care facilities early on in the pandemic, and the mortality risk of residents was up to four times higher during the pandemic compared to the previous years [15]. People residing in these facilities are often old, frail, and suffer from multiple chronic NCDs but during the first wave of the pandemic, they were often unable to leave the facilities to receive care in specialized settings. Consequently, onsite HCPs and GPs took an increasing role in managing residents’ NCDs, and support staff in these facilities took on an increasingly larger role in NCD care. Further, some persons living in the community were hesitant to visit hospitals even if they needed medical attention due to fear of infection [10].

#### Solutions proposed by the Advisory Board

As we move into the next phases of the pandemic it is essential that health services and HCPs develop specific, multidisciplinary guidelines for NCD patients, including those with specific diseases and symptoms, as well as older patients with clinically complex conditions such as frailty or multimorbidity (the presence of multiple somatic disorders in one individual) and their informal carers. This could include adapting current disease-specific guidelines so that they contain recommendations focusing on the different scenarios that are likely to occur in the near future including (i) periods of lockdown when non-urgent NCD care is scaled back; (ii) intervals when strict lockdowns have been eased but there is still a risk of contracting SARS-CoV-2, when NCD patients might be recommended to shield or reduce face-to-face contact with others and; (iii) future periods when rates of SARS-CoV-2 reduce if vaccination programmes are successful long-term on a large scale. It is important to also consider that such guidelines will be based on current knowledge and that the pandemic has hampered many research activities on NCDs (e.g., due to reallocation of resources, delays in ethical review, halting of non-COVID-19 research projects etc.).

#### The role of multistakeholder partnerships

As many countries are currently moving into new peak waves with high rates of new COVID-19 cases, it is important to establish research proposals that are specifically focused on increasing evidence-based information on the best methods for multidisciplinary NCD management during lockdown periods by measuring the short- and long-term effects of different healthcare management strategies*.* Multistakeholder partnerships can play a vital role in achieving the above-mentioned goals. First, they can provide common platforms for policymakers and HCPs to work together to provide practical, timely guidelines, through virtual conferences, advisory boards, and working groups. These events can be used to collect the perspectives of multiple stakeholders and promote opportunities for collaborative research partnerships. Further, they can support the development of guidelines co-written by multiple stakeholders through unconditional, independent financial support. An essential element of multistakeholder partnerships is the unique prospects for researchers and HCPs to collaborate with patient advocacy groups and policymakers to ensure that every perspective is heard and to help facilitate the harmonization and dissemination of scientific evidence through structured collaboration and consistent dissemination programs.

### Integrated care

#### Barriers and issues

Due to the scale-back of non-urgent care and the reduction in appointments for NCD monitoring and management, there was a shift from hospital-based NCD care to more community-level care and self-management during the first wave of the pandemic. This is likely to have had important implications for integrated care. Integrated care is an essential strategy in chronic care models [16–18] and a crucial part of management for complex NCD patients, especially older patients and those with multimorbidity [17]. It involves coordinated, collaborative care from different administrative, organizational, service delivery, and clinical levels, to ensure comprehensive and multidimensional treatment and care to patients. For patients with multimorbidity, regular comprehensive assessment from a multidisciplinary, coordinated team is a recommended approach [17, 19] but it is likely that this component was greatly disrupted during the COVID-19 pandemic. It is also important to consider the needs of informal carers in these assessments in order to prevent exhaustion, negative health and social outcomes on their part as well as low-quality daily care. Many specialized care services were cancelled and, due to the risk of SARS-CoV-2 infection, it is likely that patients with multimorbidity will have missed important assessments to track the progression of their NCD symptoms and disorders. Patients with new-onset multimorbidity are unlikely to have undergone comprehensive assessments to help stratify their medical and care needs, which is recommended [17]. Further, health services, especially geriatric and infectious disease specialties, were overwhelmed during the peak of the pandemic, and many specialist geriatric wards were converted into dedicated COVID-19 treatment centers. The long-term implications that these changes had on the integrated care of NCD patients is yet to be seen.

#### Solutions proposed by the Advisory Board

The Advisory Board members proposed numerous solutions for reshaping integrated care processes in the future. First, in the ongoing waves of the pandemic, the system needs to be reshaped by decentralizing care and shifting more NCD care into the community, with a focus on both short- and long-term solutions. It was also recommended that there should be more focus on patient self-support, aided by HCPs and informal carers. Indeed, one of the cornerstones of some integrated care models is the training of HCPs to tailor self-management support based on patient preferences and competencies and providing options for patients and families to improve self-management [17]. Therefore, a temporary shift that puts more emphasis on these components of integrated care models might help to compensate for the reduction in hospital-based NCD management during the pandemic. Similarly, other essential components of integrated care such as the use of patient-operated technology allowing patients to send information to their HCPs [17] and self-manage their disease and symptoms may need to be prioritized. However, as discussed later, patient preferences, regulations, and reimbursement for digital health solutions need to be considered. EU Institutions and Member States should also recognize and support (for instance, promoting the adoption of a common legislative framework, in the context of the European Pillar of Social Rights) the value of formal and informal care, that involves a multitude of complex activities provided also by non-professional carers. Further, it was emphasized that there needs to be an increase in the use of “case managers” or “care coordinators” (e.g., persons allocated as the active coordinator of care for an individual patient). Case managers need specific extra training to help NCD patients and their carers access reliable health information, care services, and NCD support during the pandemic. Case managers could become the point-of-call for older NCD patients, in terms of directing them to trusted healthcare information resources and supporting self-care and access to digital health solutions.

#### The role of multistakeholder partnerships

Multistakeholder partnerships may be key players who can support the development and implementation of tools, mainly digital, that can help prevent patients from having direct physical contact with HCPs while still receiving high-quality healthcare services. Importantly, they can help to assess the perspectives, needs, and preferences of patients and carers for different solutions and bring together digital health technology creators and patient organizations so that they can develop effective and user-friendly alternatives to face-to-face care that take into account the goals and capabilities of both patients and HCPs. Stakeholders need to discuss the critical issues related to the systematic design, delivery, and deployment of solutions. For example, clear criteria need to be developed to evaluate “high quality” digital health technology, the guidelines for delivery to patients (data privacy/security, accuracy of the information, user engagement, and ultimately proof of effectiveness), and guidance for HCPs in the recommendation of digital health services to patients.

### Health information

#### Barriers and issues

The COVID-19 pandemic triggered a surge in infodemics, where technology and social media have been used to provide productive and useful information to people on a large scale while also disseminating an excess of misinformation and disinformation that can potentially harm individuals. Although the primary source of lack of information and misinformation was about COVID-19, individuals also struggled with inadequate information on how to manage NCDs without direct support from HCPs (or with only remote support). Further, there were numerous problems in long-term care facilities during the first wave of the pandemic because there was a lack of knowledge and training among staff on how to reduce the risk of SARS-CoV-2 infection in residents or how to manage preexisting health problems and NCD symptoms in older and frail individuals, whose usual NCD care was disrupted due to infection control measures.

#### Solutions proposed by the Advisory Board

The Advisory Board members outlined some specific strategies to address this lack of reliable information and confusion over where to access accurate and useful information relating to NCD care. There is an urgent need to produce tools to guide people to find appropriate, trustworthy scientific and evidence-based information to improve patient empowerment. The tools should clearly help patients to assess the reliability and scientific robustness of the information they find, through the endorsement of patient advocacy groups or medical institutions. These should include both online and offline alternatives to reduce inequalities in digital health literacy. It was suggested that “best practice” patient guidebooks could be developed as well as information platforms with reliable and engaging information sources, guiding people to scientific information written in layman terms.

#### The role of multistakeholder partnerships

Multistakeholder partnerships will have a crucial role because they can support the creation of information platforms for patients to see reliable information, led by independent experts, which can be authorized by patient advocacy groups and endorsed by medical associations or institutions. Private industry members can provide sponsorship to HCPs and researchers to develop information and web-based resources that patient advocacy groups can assess, endorse, and advertise where appropriate. Similarly, support could be provided to develop online training programs for staff in long-term care facilities, with expert advice from researchers and specialized physicians, supported independently by private industry funding.

### Digital health solutions

#### Barriers and issues

One of the most noticeable changes in NCD healthcare delivery during the first wave of the pandemic was the surge in digital health solutions that were used to replace or compliment face-to-face care. Due to the cancellation and postponement of many outpatient services and a reduction in non-urgent medical appointments, many patients and HCPs had no choice but to use digital health tools to communicate with each other, particularly telemedicine and video consultations. The pandemic created an urgent need for coordinated mechanisms to respond to the outbreak across health sectors; digital health solutions may be promising approaches to address this challenge. A major problem was that these solutions were often not standardized and largely unregulated. Many European countries did not have sophisticated telehealth systems in place, although there were large differences between countries. Importantly, there were no clear policies regarding which type of patient should receive digital health solutions or who should be prioritized. Unclarity over billing, costs, and incentives for using digital health services were also relevant. Concerns over privacy and confidentially arose among patients and HCPs. Further, many HCPs, patients, and informal carers were unprepared to use many digital health solutions because they lacked training or experience. There were also inequalities in access to technological solutions. For example, some patients such as those with cognitive or sensory impairment may have had difficulties in using some tools.

#### Solutions proposed by the Advisory Board

The Advisory Board members highlighted that the pandemic could be an opportunity to trigger an increase in the development and use of digital health tools in NCD care that could be carried forward to the future. Due to the speed with which HCPs and patients have had to find alternatives to face-to-face healthcare, a priority is to adapt already existing tools and modify them to different contexts and types of patients. Importantly, all end-users, including HCPs, patients, and carers should be involved in co-developing and modifying telemedicine solutions, to ensure that their capabilities, experience, and preferences are considered. Alongside this, healthcare systems need to devise ways to improve digital health literacy via training programs and educational tools that focus on specific patient groups and people with particular care needs. Close collaboration with policymakers is needed to ensure that clear standards of care for technological health solutions are defined for pandemic periods and future waves, as well as the post-COVID-19 future.

#### The role of multistakeholder partnerships

Multistakeholder partnerships can play an essential role in aiding this process. First, they can provide an organizational structure and competences to foster the development of innovative solutions by pulling together key private and public stakeholders. They can also help to define outcomes and support research on the efficacy of such tools from multiple perspectives (e.g., disease management goals, patient preferences, healthcare costs, etc.) to validate, harmonize, and conduct cost-effectiveness analyses to ensure that new technologies truly generate an added value for the community. Although there are some existing technology platforms that score and promote apps that meet established criteria (e.g. https://www.nhs.uk/apps-library/), the rating systems used often do not feature reviews from patient advocacy organizations. These organizations can collaborate with multistakeholder partnerships to incentivize the use of effective solutions in HCPs, patients, and informal carers and motivate patients to undergo training and use technologies. These could take the form of best practice guidelines, written in layman terms, and endorsed by patient advocacy groups, outlining which digital health tools meet the right standards of safety and usability for NCD patients to complement face-to-face care and providing information on how and where to receive training and support. Further multistakeholder partnerships can be leveraged to create communication hubs to increase interaction between different care levels and settings, for example by improving communication between primary and secondary care and long-term care facilities through discussion forums and information exchange platforms.

## Relevance and conclusions

The above-mentioned issues have important implications for the therapeutic paradigm and, in particular, treatment adherence, both in terms of pharmaceutical and non-pharmaceutical interventions. Poor medication adherence to NCD drugs is likely to have increased because of the pandemic due to multiple reasons, including lack of medications, reduced access to pharmacies, and a decrease in HCP services for medication control and adjustment. Non-pharmaceutical treatments including cognitive training or physiotherapy have also been affected, as well as social care and home support, which is an essential component of integrated care for older patients with multimorbidity [17]. COVID-19 vaccination programs are currently ongoing and new waves of infection are already occurring in numerous countries, yet it is unlikely that the majority of the world’s population will have access to vaccinations in the intermediate future. Moreover, logistic issues may compromise or delay access to vaccinations especially in low-income countries where a lack of good infrastructures may create a barrier to distribution. These settings may also face issues in financing and implementing measures to overcome disruptions to NCD care, thus creating health inequities. It is highly likely that there will be new, repeated lockdowns and other measures to control SARS-CoV-2 infection; this highlights the need for permanent changes to NCD care and health service structures, that could prevent both the acute phase impact and the long-wave of chronic burden in the years to come. Harmonized management guidelines and clinical protocols can, on one side, strengthen the collaboration among institutions and, on the other side, provide an important resource for HCPs. Even before the pandemic, the shift from a centralized health service provision to integrated services involving different care levels was a priority to improve accessibility while safeguarding system sustainability; the COVID-19 emergency further pushed this opportunity, highlighting the need for infrastructures and competences that can address chronic patients' needs at the community level in a manner that is coordinated with specialized settings.

Indeed, in September 2020, a joint statement was issued by WHO, UN, UNICEF, UNDP, UNESCO, UNAIDS, ITU, UN Global Pulse, and IFRC (20), which called all stakeholders “to collaborate with the UN system, with Member States and with each other, and to further strengthen their actions to disseminate accurate information and prevent the spread of mis- and disinformation.”

The COVID-19 pandemic has been an unprecedented disruptive event, but important lessons can be learned to help better improve NCD care in the future. Synergies between private and public stakeholders, collaborating institutions, and patient advocacy groups may help to promote swift changes to NCD prevention and patient care to cope with the ongoing pandemic. We need to ensure that there are effective methods to increase communication between all stakeholders and involve all players in the development of clinical guidelines and digital health tools, healthcare restructuring, and patient support in the short-, medium- and long-term future. Partnership models will play an essential role in achieving these goals. Accordingly, a strategic investment in better NCD management is not only beneficial for the prevention of deterioration and related NCD complications but also reduces the impact of pandemics and other health threats on health and social care systems as well as economies in general. Hence, the recommendations proposed contribute to a healthy and more resilient and sustainable society.

## Data Availability

Not applicable.
